# The biological interplay between aortic stenosis and transthyretin cardiac amyloidosis

**DOI:** 10.1093/ehjcr/ytag618

**Published:** 2026-08-31

**Authors:** Takeshi Kitai, Kitae Kim, Piotr Nikodem Rudzinski

**Affiliations:** Department of Heart Failure and Transplantation, National Cerebral and Cardiovascular Centre, 6-1 Kishibe-Shinmachi, Suita, Osaka 564-8565, Japan; Structural Heart Program, St. Michael’s Hospital, University of Toronto, Toronto, 30 Bond Street, Toronto, Ontario M5B 1W8, Canada; Department of Coronary and Structural Heart Diseases, The Cardinal Stefan Wyszyński, National Institute of Cardiology, Warsaw, Alpejska 42, 04-628 Warsaw, Poland

**This editorial refers to ‘Challenges in diagnosis and management of severe aortic stenosis with coexisting cardiac amyloidosis: a case report’, by R. Çetin Güvenç *et al.,*  https://doi.org/10.1093/ehjcr/ytag511.**

Aortic stenosis (AS) and transthyretin cardiac amyloidosis (ATTR-CM) are increasingly recognized as coexisting diseases in older adults.^1^ AS is a common age-related valvular disease, with its prevalence increasing markedly with advancing age and approaching 10% among individuals aged 80 years or older.^2,3^ Contemporary studies have demonstrated that ATTR-CM is present in ∼10%–15% of elderly patients undergoing transcatheter aortic valve intervention and is associated with adverse clinical outcomes. While this overlap has traditionally been regarded as a simple consequence of ageing, accumulating evidence suggests that ATTR-CM and AS share common biological pathways and engage in a complex biological interplay that may reciprocally influence disease progression, rather than representing merely age-related bystander diseases.^4^ This evolving concept provides a new framework for understanding the coexistence of AS and ATTR-CM and has important implications for diagnosis, therapeutic decision-making, and future research.

While the underlying mechanisms remain incompletely understood, a pathophysiological link has been proposed whereby transthyretin amyloid deposition within the aortic valve may contribute to valvular degeneration and the development of AS.^5^ Although definitive mechanistic evidence remains limited, these observations support the concept that the coexistence of AS and ATTR-CM may reflect more than shared ageing alone. From a clinical perspective, timely diagnosis of ATTR-CM is crucial, as early initiation of TTR-targeted therapy has the potential to modify the natural history of the disease, improve survival, and reduce heart failure hospitalizations.^6^ However, the diagnosis of ATTR-CM can be particularly challenging in patients with severe AS, as both conditions share overlapping clinical features, including heart failure symptoms and concentric left ventricular (LV) hypertrophy. These findings are often attributed to AS alone, resulting in under diagnosis and delayed treatment of ATTR-CM.

In the current issue of *European Heart Journal Case Reports*, Güvenç *et al.*, reported an 85-year-old woman with symptomatic severe AS who was ultimately diagnosed with concomitant wild-type ATTR-CM.^7^ The patient presented with several clinical red flags, including atrial fibrillation, a pseudo-infarction pattern on electrocardiography, disproportionate LV hypertrophy, and an apical sparing pattern on echocardiography, prompting further evaluation that confirmed ATTR-CM. Following successful transcatheter aortic valve implantation, TTR-targeted therapy was initiated. Rather than representing an exceptional case, this report illustrates a typical clinical scenario in which careful recognition of multiple red flags enables timely diagnosis and comprehensive management of patients with concomitant AS and ATTR-CM. Clinically, this biological and phenotypic overlap explains why concomitant ATTR-CM is often overlooked in patients with severe AS. Although individual red flags such as atrial fibrillation, conduction disease, increased LV wall thickness, apical sparing, elevated natriuretic peptides, or low-grade troponin elevation are not specific, the simultaneous presence of multiple findings should prompt evaluation for ATTR-CM using bone scintigraphy and monoclonal protein testing. This clinical overlap extends beyond diagnostic challenges and may reflect a deeper biological relationship between AS and ATTR-CM. Emerging pathological evidence suggests that these diseases are biologically interconnected rather than simply coexisting age-related conditions.

## Biological interplay between AS and ATTR-CM

Recent pathological studies have provided novel insights into the potential biological interaction between AS and ATTR-CM. Calcific aortic valve disease is now recognized as an active biological process characterized by chronic inflammation, extracellular matrix remodelling, myofibroblast activation, and osteogenic differentiation of valve interstitial cells, rather than passive age-related degeneration. These processes drive progressive valve calcification and leaflet stiffening, ultimately resulting in severe AS.

More recently, Sud *et al*.^5^ summarized accumulating pathological and experimental evidence suggesting that amyloid deposition may represent an additional component of this pathological cascade. Histopathological studies have demonstrated that apolipoprotein-related amyloid deposits are preferentially localized adjacent to calcified regions within stenotic aortic valves. Experimental evidence further suggests that these deposits may amplify local inflammatory signalling, extracellular matrix remodelling, and osteogenic differentiation of valve interstitial cells, potentially accelerating valve calcification rather than representing an incidental age-related finding.

**Figure 1:**
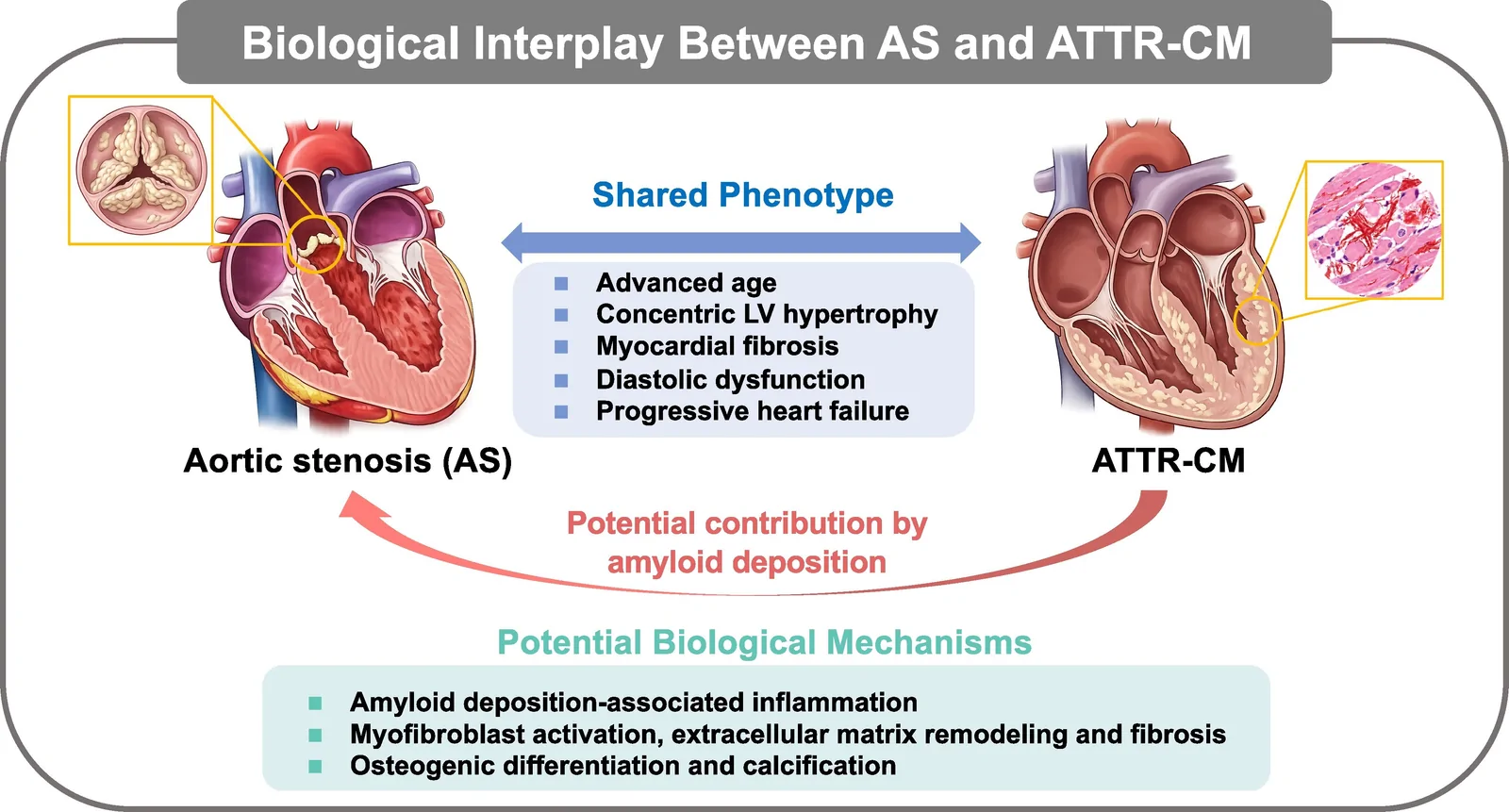
Biological interplay and shared pathophysiological pathways between Aortic Stenosis and Transthyretin Cardiac Amyloidosis. Aortic Stenosis and Transthyretin Cardiac Amyloidosis are increasingly recognized as biologically interconnected rather than merely coexisting age-related diseases. Both conditions share overlapping clinical phenotypes, including advanced age, concentric left ventricular hypertrophy, myocardial fibrosis, diastolic dysfunction, and progressive heart failure. On a molecular level, amyloid deposition may contribute to chronic inflammation, extracellular matrix remodelling, and osteogenic differentiation, potentially accelerating valvular calcification and disease progression when AS and ATTR-CM coexist. AS, aortic stenosis; ATTR-CM, transthyretin amyloid cardiomyopathy.

Although a direct causal relationship between valvular amyloid deposition and AS progression remains unproven, these observations support a broader concept that amyloid deposition, inflammation, fibrosis, and calcification are biologically interconnected processes. These observations provide biological plausibility for the frequent coexistence of AS and ATTR-CM and support the hypothesis that these diseases may interact through shared pathological pathways. Chronic pressure overload in AS promotes hypertrophy and interstitial fibrosis, whereas ATTR-CM causes extracellular amyloid deposition with progressive myocardial stiffening. Once these processes coexist, they may synergistically worsen ventricular compliance, diastolic dysfunction, and heart failure. The proposed biological interactions between AS and ATTR-CM are summarized in the *Figure 1*.

## Therapeutic implications

If AS and ATTR-CM are viewed as biologically interconnected rather than independent age-related diseases, therapeutic strategies should also target both disease processes. Although these diseases have distinct primary mechanisms, both contribute to progressive myocardial dysfunction and poor prognosis if left untreated.^8,9^ In a prospective cohort of 407 patients referred for TAVI, concomitant ATTR-CM was identified in 12% of patients and was associated with increased 1-year mortality. However, patients with concomitant ATTR-CM who underwent TAVI had significantly better outcomes than those managed conservatively, and their survival was comparable to that of patients with isolated AS. These findings suggest that the presence of ATTR-CM alone should not preclude aortic valve intervention, and TAVI should remain the standard treatment for severe AS when clinically indicated.

A recent multicentre registry evaluated 226 patients with ATTR-CM and moderate or severe AS (87% had severe AS), of whom 72% underwent TAVI, 4% underwent surgical aortic valve replacement (AVR), and 24% received conservative management.^10^ TTR-targeted therapy, predominantly tafamidis (99%), was prescribed to 69 patients (31%). Both AVR and TTR-targeted therapy were independently associated with improved survival (HR 0.42, 95% CI 0.26–0.70, and HR 0.40, 95% CI 0.24–0.68, respectively), and patients receiving both therapies had the most favourable prognosis. Although these findings were derived from a non-randomized study and should be interpreted with caution because of potential baseline differences, TTR-targeted therapy should be considered after TAVI, in patients with preserved functional status and a reasonable life expectancy, given the established benefits of disease-modifying therapy for ATTR-CM, including tafamidis, acoramidis and vutrisiran, on both quality of life, heart failure hospitalization, and survivals.^6^

However, there is currently no evidence that TTR-targeted therapies for ATTR-CM modify the progression of AS *per se*. Although histopathological studies have suggested an association between amyloid deposition and valvular calcification, the precise biological mechanisms underlying this association remain incompletely understood. A recent case series using computed tomography demonstrated lower-than-expected aortic valve calcium scores in patients with severe AS and ATTR-CM,^11^ suggesting that pathological mechanisms beyond valvular calcification may also contribute to the development and progression of AS in these patients. These unresolved questions warrant further investigation and may ultimately shape the next generation of therapeutic strategies for patients with concomitant AS and ATTR-CM.

## Conclusion

The coexistence of AS and ATTR-CM should no longer be regarded simply as an age-related coincidence but rather as a biologically interconnected disease process. Emerging evidence supports the concept that these diseases share common biological mechanisms and may reciprocally influence myocardial remodelling and disease progression. Recognizing this interaction not only facilitates earlier diagnosis but also provides a rationale for integrated therapeutic strategies targeting both the valve and the myocardium. Viewing AS and ATTR-CM as a biologically interconnected dual pathology may ultimately transform both diagnostic strategies and therapeutic decision-making in an ageing population.

## Data Availability

No individual participant data were used in the preparation of this Editorial.

## Lead author biography

Takeshi Kitai, MD, PhD, FHFA, FESC, is Director of the Section of Heart Failure and Structural Heart Disease, Department of Heart Failure and Transplantation, National Cerebral and Cardio vascular Center, Japan. His clinical and research interests include cardiomyopathy, heart failure, valvular heart disease, and structural heart interventions.
